# Protein Acetylation at the Interface of Genetics, Epigenetics and Environment in Cancer

**DOI:** 10.3390/metabo11040216

**Published:** 2021-04-01

**Authors:** Mio Harachi, Kenta Masui, Webster K. Cavenee, Paul S. Mischel, Noriyuki Shibata

**Affiliations:** 1 Department of Pathology, Division of Pathological Neuroscience, Tokyo Women’s Medical University, Tokyo 162-8666, Japan; harachi.mio@twmu.ac.jp (M.H.); shibatan@twmu.ac.jp (N.S.); 2 Ludwig Institute for Cancer Research, University of California San Diego, La Jolla, CA 92093, USA; wcavenee@health.ucsd.edu; 3 Department of Pathology, Stanford University School of Medicine, Stanford, CA 94305, USA; pmischel@stanford.edu

**Keywords:** metabolic reprogramming, microenvironment, protein acetylation, epigenetics, mechanistic target of rapamycin (mTOR) complexes

## Abstract

Metabolic reprogramming is an emerging hallmark of cancer and is driven by abnormalities of oncogenes and tumor suppressors. Accelerated metabolism causes cancer cell aggression through the dysregulation of rate-limiting metabolic enzymes as well as by facilitating the production of intermediary metabolites. However, the mechanisms by which a shift in the metabolic landscape reshapes the intracellular signaling to promote the survival of cancer cells remain to be clarified. Recent high-resolution mass spectrometry-based proteomic analyses have spotlighted that, unexpectedly, lysine residues of numerous cytosolic as well as nuclear proteins are acetylated and that this modification modulates protein activity, sublocalization and stability, with profound impact on cellular function. More importantly, cancer cells exploit acetylation as a post-translational protein for microenvironmental adaptation, nominating it as a means for dynamic modulation of the phenotypes of cancer cells at the interface between genetics and environments. The objectives of this review were to describe the functional implications of protein lysine acetylation in cancer biology by examining recent evidence that implicates oncogenic signaling as a strong driver of protein acetylation, which might be exploitable for novel therapeutic strategies against cancer.

## 1. Introduction

Metabolic reprogramming is an emerging hallmark of cancer and is driven by abnormalities of oncogenes and tumor suppressors [1]. Accelerated metabolism translates into cancer cell aggression through the dysregulation of rate-limiting metabolic enzymes as well as by facilitating the production of intermediary metabolites [2], but it remains to be fully clarified how a shift in the metabolic landscape reshapes the intracellular signaling to promote the survival of cancer cells [3,4]. Post-translational modification (PTM) of proteins is an essential phenomenon that dynamically regulates cellular functions in an appropriate spatio-temporal manner and which is responsive to a drastic shift in the microenvironment [5,6]. Recent studies have demonstrated that lysine acetylation, one of the major protein PTMs, is prevalent for a variety of enzymes that catalyze intracellular metabolism [7], suggesting that protein acetylation plays a major role in cellular functions including synchronous metabolic cascades [7,8]. Advanced proteomics using mass spectrometry have further enabled the global identification and characterization of thousands of acetylation sites which are involved in the regulation of protein function by affecting protein interactions with nucleic acids and other proteins, the catalytic activity of proteins, and protein sublocalization [8,9]. More importantly, cancer cells exploit the PTM of proteins with acetylation for adapting to the microenvironment, suggesting that acetylation may dynamically modulate the phenotypes of cancer cells at the interface of genetics and the environment [5,10,11,12]. Here, the purpose of the review carried out was to describe the functional implications of protein lysine acetylation in different cellular compartments, highlighting its biological role and prognostic value in cancer, by evaluating recent evidence implicating oncogenic signaling as a strong driver of protein acetylation, which could be exploitable for novel therapeutic strategies against cancer.

## 2. Regulatory Mode of Protein Lysine Acetylation

### 2.1. Acetylation Enzymes: Writers, Erasers and Readers of Lysine Acetylation

#### 2.1.1. Writers of Lysine Acetylation

**Figure 1:**
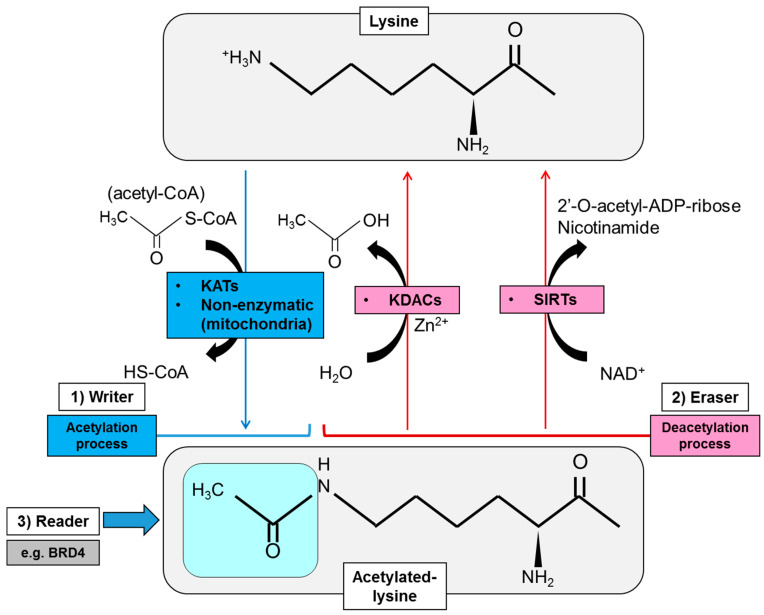
Regulatory mode of protein lysine acetylation by modification enzymes including writers, erasers and readers of lysine acetylation. Writers of protein acetylation (KATs) transfer the acetyl group from an intermediary metabolite acetyl-CoA to the epsilon NH3^+^ side chain of lysines of the targeted protein. Acetylation in mitochondrial protein can also be processed non-enzymatically. Acetylation eraser (deacetylase) activity is mediated by Zn^2+^-dependent KDACs [class I, II and IV histone deacetylases (HDACs)], and class III HDAC or sirtuins (SIRTs) depending on NAD^+^. The acetylation marks on lysine residues of the histone protein are read by small protein modules called the bromodomain and extra-terminal (BET) proteins including BRD4. ADP, adenosine diphosphate; BRD4, bromodomain-containing protein 4; KAT, lysine acetyltransferase; KDAC, lysine deacetylase; NAD, nicotinamide adenine dinucleotide; SIRT, sirtuin.

Protein acetylation, represented by the status of N-epsilon acetyl lysine, is controlled by the combinatory action of both lysine acetyltransferases (KATs) and lysine deacetylases (KDACs) [13]. KATs transfer the acetyl group from the intermediary metabolite, acetyl coenzyme A (acetyl-CoA), to the epsilon NH_3_^+^ side chain of lysines within the targeted protein, that is to say, work as a “writer of lysine acetylation” (Figure 1). The transfer of the acetyl group eventually neutralizes the positive charge on the lysine residue and changes the structure of the R group on the amino acid, affecting multifaceted aspects of the targeted protein [14]. An array of mammalian proteins have been reported to possess endogenous KAT activity, but KATs are basically subclassified into three families based on phylogenetic sequence similarities: (1) the GNAT (GCN5-related *N*-acetyltransferases) family that includes GCN5 (KAT2A) and PCAF (p300/CBP-associated factor, KAT2B), (2) the p300 (KAT3A)/CBP (CREBBP) [cAMP-responsive element-binding protein (CREB)-binding protein, KAT3B] family, and (3) the MYST family for MOZ (monocytic leukemia zinc finger protein, KAT6A), Ybf2, Sas2 and Tip60 (KAT5) (Table 1) [15]. KATs are usually incorporated within the unique molecular complexes that increase capacity for target specificity and facilitate interactions with a range of other proteins at a subset of enhancer and promoter elements as well as in gene bodies of transcriptionally active genes [14,15]. Of interest, the sublocalization and enzymatic activity of KATs can also be regulated by PTMs that include phosphorylation and acetylation, and in the example of p300 and PCAF, can be auto-acetylated for activation and stability, suggesting the intricate reciprocal interaction among the subcellular components by acetylation [16]. Importantly, some of these regulatory mechanisms of KATs rely upon oncogene activation, and it is not surprising that several KATs are associated with oncogenesis, in addition to other important functions in cellular differentiation and embryonic development [17].

Lysine acetylation can also be achieved in a non-enzymatic fashion (Figure 1), notably in the acetylation of calf thymus nuclear histone protein with acetyl-CoA, that is dependent on pH, period of incubation, ionic strength and ionic species [35]. Recent studies also suggested that, under basal conditions, “non-enzymatic lysine acetylation” is maintained at a very low stoichiometry in mitochondria [36,37]. Acetyl-CoA and acetyl-glutathione reversibly acetylate protein cysteine residues, and non-enzymatic N-acetylation of lysine residues by acetyl-CoA occurs via proximal S-acetylated thiol intermediates that are sensitive to glyoxalase II in mitochondria [36]. Further, a large-scale lysine acetylation study has revealed that only approximately 20% of non-enzymatic acetylation sites are targeted by mitochondrial protein deacetylases, and there are sites at which acetylation does not alter the activity of the protein, while others can irreversibly inhibit the targeted enzyme [38]. Currently, less is known about the role of non-enzymatic acetylation in cancer in comparison with that by KATs, but several lines of evidence suggest that targeting non-enzymatic acetylation could be an important niche for future development of therapies in targeting cancer metabolism [39].

#### 2.1.2. Erasers of Lysine Acetylation

Erasers of protein lysine acetylation are a group of protein called KDACs or HDACs (histone deacetylases) because this group mainly deacetylates the epsilon-amino group of lysine (K) residues on the nuclear histone (H) proteins that constitute the core octamers for nucleosomal complexes (Figure 1). Histone deacetylation results in the restoration of their positive charge, which increases their ability to bind to negatively charged DNA and eventually hinder the access of transcriptional complexes. In mammalian HDACs, 18 highly conserved genes were noted [40], and they are subdivided into Class I (HDAC1, HDAC2, HDAC3, HDAC8), Class IIa (HDAC4, HDAC5, HDAC7, HDAC9), Class IIb (HDAC 6, HDAC 10), Class III (sirtuin, or SIRT1-7) and Class IV (HDAC11) on the basis of phylogenetic analysis and sequence similarity to yeast factors (Table 2) [41]. The catalytic domain of HDACs is similar to a pocket and consists of two adjacent histidine residues, two aspartate residues and one tyrosine residue with Zn^2+^ ions as the core [42], whereas the deacetylase activity of class III or sirtuins (SIRTs) depends on nicotinamide adenine dinucleotide (NAD^+^) rather than on Zn^2+^-dependent enzymes (Figure 1) [43]. Similar to KATs, HDACs act on multiprotein complexes which ensure specific mechanisms of active repression of transcription in the promoter regions of the gene [44]. Notably, HDACs were also reported to deacetylate non-histone nuclear/cytoplasmic protein to regulate the sublocalization and activity of the targeted protein, which is involved in physiological, as well as abnormal, conditions including metabolic diseases and cancer [45,46,47].

#### 2.1.3. Readers of Lysine Acetylation

One of the primary proteins that are targeted by KAT/HAT (histone acetyltransferase) and KDAC/HDAC are histone proteins, whose dynamic modification is involved in various biological processes and is correlated with several human diseases, including cancer. The acetylation marks on lysine residues of the histone proteins are read by small protein modules called the bromodomain and extra-terminal (BET) proteins (namely BRD2, BRD3, BRD4, and BRDT) that utilize tandem bromodomain (BRD) modules to recognize and dock themselves on the acetylated lysines (Figure 1) [68]. BET protein BRD4 binds particularly to acetylated histones at enhancers and promoters via its bromodomains where it regulates transcriptional elongation through interaction with transcriptional complexes including P-TEFb (positive transcription elongation factor b) and mediator [69]. Of interest, BRD4 is highly enriched in super-enhancers that drive the expression of oncogenic transcription factors such as c-Myc, suggesting that targeting BET family proteins could be a promising approach for cancer treatment [70]. Additionally, a recent report suggests a mechanism by which enhancer-directed transcripts (eRNAs) are directly associated with gene regulation by modulating enhancer interactions and transcriptional functions of BRD4 [71], further expanding the role of BET proteins in the normal physiology, as well as cancer biology. Together with the writers and erasers of protein lysine acetylation, readers of lysine acetylation thus are of equal importance for the phenotypes of cancer cells and provide the promising opportunity to develop a novel type of therapeutics to specifically target cancer transcriptomes.

### 2.2. Donor Substrate for Acetylation: Production of Intermediary Metabolites for Protein Acetylation

**Figure 2:**
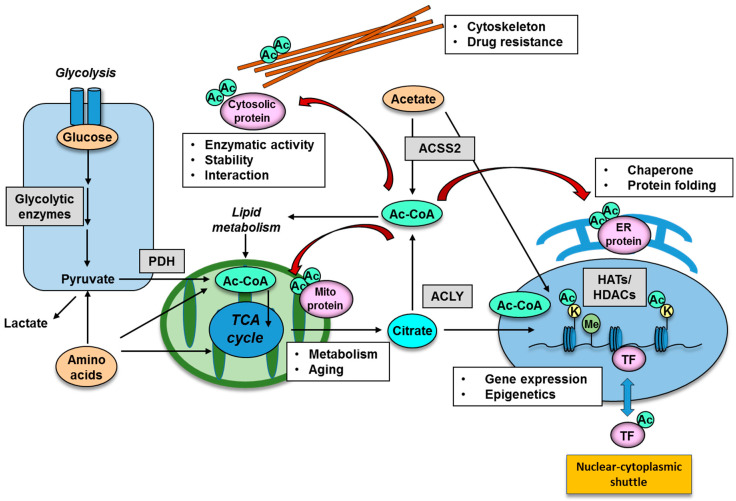
Functional significance of lysine acetylation in different cellular organelle. Protein acetylation is mediated by an intermediary metabolite acetyl-CoA, produced by the enzymes of PDH, ACLY and ACSS2. Representative nuclear protein which could be regulated by acetylation is histone protein and transcription factors, the acetylation of which has an impact on gene expression and epigenetic changes. Acetylation of cytoplasmic organelle is represented by that of cytoskeleton, mitochondria and ER protein, and acetylation in these organelle could be involved in intracellular metabolism, aging, protein chaperone and drug resistance. Ac, acetyl group; Ac-CoA, acetyl-CoA; ACLY, ATP citrate lyase; ACSS2, acyl-coenzyme A synthetase short-chain family member 2; ER, endoplasmic reticulum; HAT, histone acetyltransferase; HDAC, histone deacetylase; K, lysine residue; Me, methyl group; PDH, pyruvate dehydrogenase; TF, transcription factor.

Many enzymes that play important roles in epigenetic gene regulation harness intermediary metabolites as co-substrates yielded by cellular metabolic reprogramming [1,72]. The methyl-donor SAM (S-adenosylmethionine) which is derived from methionine is utilized by methyltransferases, and its metabolism can profoundly affect epigenetic changes including DNA and histone methylation status [73,74]. As for protein acetylation, acetyl-CoA is the substrate used to modify histone tails as well as non-histone proteins and can be produced through a variety of metabolic pathways [75]. The acetyl donor, acetyl-CoA, can be obtained from a number of sources, primarily through the conversion of pyruvate from glycolysis and citrate from the tricarboxylic acid (TCA) cycle. Acetyl-CoA is also released from the breakdown of fatty acids and amino acids in the mitochondria while pyruvate derived from glucose can be converted into acetyl-CoA by the pyruvate dehydrogenase complex (PDC) (Figure 2). In addition, recent studies have demonstrated that the dynamic translocation of mitochondrial pyruvate dehydrogenase (PDH) to the nucleus provides a pathway for nuclear acetyl-CoA synthesis required for histone acetylation and epigenetic regulation [76,77,78]. Importantly, the nuclear translocation of PDH is facilitated by the stimulation of growth factor receptor and mTOR (mechanistic target of rapamycin) pathway signaling [77]. ATP citrate lyase (ACLY) is another key enzyme responsible for generating cytosolic acetyl-CoA and oxaloacetate which are important metabolites for cancer cells (Figure 2). ACLY can be regulated by growth factor stimulation, which is also required for histone acetylation and gene expression [79], and inhibition of ACLY results in tumor growth arrest [80]. Of interest, acetyl-CoA can be produced by PDH from glucose and by acyl-coenzyme A synthetase short-chain family member 2 (ACSS2) from acetate, both of which are closely associated with the biology of the malignant brain tumor, glioblastoma (GBM), through acetylation of cytosolic proteins (Figure 2) [81]. In addition to glucose, acetate is also an emerging target nutrient of interest for cancer biology. Acetate can be used by tumor cells as an important bioenergetic fuel or as a nutritional source to support lipid biosynthesis as well as a precursor for acetylation of histones and other proteins and hence can serve as an epigenetic and post-translational modifier [82]. Of note, despite its low circulating concentration in plasma, acetate could still exert its effects through intra- and intercellular recycling of acetate molecules within the tumor microenvironment, leading to the role of acetate as a positron emission tomography (PET) imaging probe for cancer as well as an exploitable metabolite for future anti-ACSS2 therapy against cancer [83].

## 3. Functional Significance of Lysine Acetylation in Different Cellular Organelle

### 3.1. Acetylation of Nuclear Proteins: Implication for Epigenetics

One of the essential constituents in the nucleosomal structure are the histone proteins, where their N-terminal tails can undergo a variety of posttranslational covalent modifications including methylation, acetylation, ubiquitination, sumoylation and phosphorylation on specific residues [84]. These modifications eventually affect regional or whole chromatin structure and regulate key biological processes such as transcription, replication and repair, leading to either promotion or suppression of gene expression, depending upon the spatio-temporal patterns of the modification [84]. For example, lysine acetylation is correlated with transcriptional activation, whereas lysine methylation results in transcriptional activation or repression depending upon the modified residue species and the degree (i.e., mono-, di-, tri-) of methylation [85]. Histone H3 acetylation at the lysine 27 residue (H3K27ac) locates to the promoter and enhancer of the gene in specific loci and H3K27ac modification has a key role in regulating genome conformation by establishing TADs (topologically associating domains) which fold genome DNA into separate domains with specific functions [86]. Enhancer regions are often primed by the monomethylation of histone H3 at lysine 4 (H3K4me1) mark and are fully activated upon deposition of H3K27ac, where DNA accessibility for transcription factors and activators can be augmented [87,88]. Conversely, enhancers are decommissioned by the release of transcription factors, which is accompanied by removal of the H3K4me1 and H3K27ac histone marks and reduced chromatin accessibility. Furthermore, a recent study demonstrated the presence of bivalent chromatin domains marked by both activating and repressive chromatin modifications which could be associated with subtype-specific signatures in developmental or neoplastic cells [89].

Histone modification patterns are dynamically regulated by enzymes that add and remove covalent modifications to histone proteins. Histone acetyltransferases (HATs) and histone methyltransferases (HMTs) add acetyl and methyl groups, whereas histone deacetylases (HDACs) and histone demethylases (HDMs) remove acetyl and methyl groups, respectively (Figure 2) [90]. Aberrant patterns of histone modifications are observed in several types of cancer and might be therapeutically exploitable [91]; for example, the heterogeneity of malignant brain tumors across the entire age spectrum was demonstrated in terms of histone modifications on the tumor epigenomic signatures [92]. In addition to histone modifications, the maintenance DNA methyltransferase (DNMT1) can be regulated through Tip60-mediated acetylation, which targets DNMT1 for proteasomal degradation [93], suggesting that DNA methylation may also be affected by nutrient status and protein PTMs.

Another example of nuclear proteins that could be regulated by acetylation are the transcription factors (Figure 2). The stability of transcription factors, such as p53, FoxO (forkhead box-containing protein, O subfamily), and c-Myc, can be affected by acetylation, achieved by blocking ubiquitination of the same residues, which then targets the protein for proteasomal degradation [94,95,96]. Acetylation of FoxO also regulates its function through altering its affinity with target DNA and its sensitivity for phosphorylation [97]. Importantly, p53, FoxO and c-Myc are transcription factors with major roles for pro- or anti-tumorigenic potential, depending on the context of tumor types, and suggests that PTM by acetylation could be associated with tumorigenesis through the regulation of oncogenic transcription factors. The function of DNA repair enzymes can also be regulated in the nucleus through PTMs. ATM (ataxia telangiectasia mutated) is a serine/threonine protein kinase that is recruited and activated by DNA double-strand breaks. It phosphorylates several key proteins that initiate activation of the DNA damage checkpoint, leading to cell cycle arrest, DNA repair or apoptosis, which is an essential mechanism of genome DNA protection. ATM kinase activity is tightly regulated by Tip60-dependent acetylation at K3016, affecting the ATM-dependent phosphorylation of p53 and CHK2 (checkpoint kinase 2) proteins, the dysregulation of which is associated with the formation of various types of cancer [98].

### 3.2. Acetylation of Cytosolic Proteins in Specific Organelles

Acetylation of non-histone, cytoplasmic proteins also has many biological implications, and non-histone acetylation plays a role in protein stability, DNA binding, gene expression, protein interactions, localization, messenger-ribonucleic acid (mRNA) stability and enzymatic activity [99]. A representative functional example for cytoplasmic protein acetylation is that of cytoskeletal components (Figure 2). α-tubulin, that together with β-tubulin forms the heterodimeric building block of microtubules, was the first cytoplasmic protein described to be acetylated [100]. Acetylation of α-tubulin, a well established marker of microtubule stability [101], is induced on lysine 40 (K40) by the α-tubulin acetyltransferase 1 (ATAT-1) [102], and can be reversed by histone deacetylase 6 (HDAC6) and sirtuin 2 (SIRT2) [103]. Of interest, acetylation of cytoskeletons is associated with cancer biology, where high HDAC6 and low levels of acetylated α-tubulin are associated with good prognosis and increased disease-free survival of breast cancer patients [104].

Interestingly, an extensive proteomic survey of cellular proteins has revealed that a large number of mitochondrial proteins are subject to reversible lysine acetylation [105]. Indeed, acetylation is an abundant modifications of mitochondrial protein: 277 acetylation sites were identified in 133 proteins, and at least 20% of all mitochondrial proteins are lysine-acetylated [105]. It is well known that three mitochondrial deacetylases (SIRT3, SIRT4, SIRT5) mediate mitochondrial protein acetylation levels. Recent reports have shown that a series of targeted proteins are involved in metabolic pathways such as the TCA cycle, oxidative phosphorylation, β-oxidation of lipids, amino acid metabolism, carbohydrate metabolism, nucleotide metabolism and the urea cycle (Figure 2) [8,106,107]. Eventually, alterations in mitochondrial acetylation states, and, hence, alterations in carbon substrate utilization, may contribute to the unusual preference for aerobic glycolysis and glutaminolysis, the emerging features frequently observed in numerous forms of cancer [108].

Other intracellular components which could be regulated by lysine acetylation are the endoplasmic reticulum (ER) and Golgi apparatus. Proteomic studies have assessed the ER acetylome, and predicted wide-ranging biological implications of this pathway [109]. The list of ER-resident proteins includes chaperones and enzymes involved with PTM and protein folding (Figure 2) [10]. ER acetylation has been reported to be catalyzed by the two ER-based KATs, AT-1 (also known as camello-like 2 and N-acetyltransferase 8B) and AT-2 (also known as camello-like 1 and N-acetyltransferase 8). Both of these members of the camello family belong to the GNAT superfamily (Table 1) [110]. Of interest, manipulation of AT-1 function in mice leads to the appearance of neurodegenerative features, inflammation and cancer [21]. Together, protein lysine acetylation appears to be an essential component of specific subcellular organelles, and its aberration could lead to cancer formation.

## 4. Aberrant Protein Acetylation in the Phenotypes of Cancer Cells

### 4.1. Driver Mutations of Lysine Acetylation/Deacetylation Genes in Cancer

Consistent with the importance of acetylation levels in cells, somatic mutations in KATs lead to malignancy, and KATs act as tumor suppressors or oncogenes in a context- and cell type-specific manner (Table 1). Mutations in cAMP-responsive element-binding protein (CREB)-binding protein (CREBBP) and EP300, that are the responsible genes for Rubinstein–Taybi syndrome of multiple congenital anomalies, could also be involved in hematological cancer including those leukemias where chromosome translocations disrupt the CREB-binding protein (CBP) gene function [24]. Pro-tumorigenic mutations in CBP and EP300 tend to be inactivating mutations, suggesting that they act as tumor suppressors [15]. The MYST family gene monocytic leukemia zinc finger protein (MOZ) was reported to be fused to CBP, discovered initially in acute myeloid leukemia (AML) [30], and chromosomal translocations in AML can also fuse MOZ to the CBP homologue p300 [111]. These findings highlight the importance of fusion of acetylation-related genes in hematological tumors. Chromosomal translocations involving MOZ appear to create bona fide oncogenes. In addition to hematological cancer, KATs mutations also contribute to tumorigenesis and solid tumor cancer stem cell (CSC) function [33]. The GNAT family PCAF missense variants with CBP truncations and intronic microdeletions have found in human epithelial cancer cell lines and primary tumors [19], and the deregulation of distinct GCN5/PCAF-containing complexes leads to the malignant transformation of the cells [18].

HDACs work as both oncogenes and tumor suppressor genes to contribute to tumorigenesis (Table 2). The frameshift mutation in exon 1 of the HDAC2 gene is largely confined to colon tumors with microsatellite instability (MSI), which produces a premature stop codon that results in loss of HDAC2 protein expression [48]. Importantly, HDAC2-deficient colon cancer cells are highly refractory to the apoptosis induced by HDAC inhibitors. Aberrations in other classes of HDACs are also associated with CSC function and tumor stratification [55,57]. Sirtuins are a particular type of HDACs, the function of which is to influence extension of lifespan (longevity). Sirtuin 2 (SIRT2) is a class III NAD^+^-dependent deacetylase, which regulates a broad range of biological functions, including aging, metabolism, differentiation, genome maintenance, and tumor suppression [43]. Of interest, naturally occurring cancer-associated SIRT2 mutations at evolutionarily conserved sites disrupt its deacetylation of DNA-damage response proteins by impairing SIRT2 catalytic activity or protein levels [60], supporting a model for SIRT2’s tumor-suppressive function which contributes to genomic stability. Additionally, SIRT family can also function as tumor suppressors, especially those residing in mitochondria, including SIRT3, SIRT4 and SIRT5 (Table 2).

As a reader of protein lysine acetylation, BRD4 is largely acknowledged in cancer for its role in super-enhancer (SE) organization and oncogene expression. Inhibition of BRD4 shortcuts the communication between SEs and target promoters with a subsequent cell-specific repression of oncogenes to which cancer cells are addicted [112]. Importantly, BRD4 itself is a target of mutation in cancer: NUT (nuclear protein in testis) carcinoma (formerly known as NUT midline carcinoma) is characterized by the presence of NUT fusion oncogenes, the most common being BRD4-NUT [113]. BRD4 genetic amplification also facilitates an oncogenic gene expression program in ovarian high-grade serous carcinomas and confers sensitivity to BET inhibitors [114]. Of interest, BRD4 inhibition induced homologous recombination deficiency (HRD) and sensitized cells across multiple tumor lineages to PARP inhibitors, regardless of BRCA1/2, TP53, RAS, or BRAF mutation status, through depletion of the DNA double-stand break resection protein CtIP (C-terminal binding protein interacting protein) [115]. All these findings indicate that lysine acetylation genes play a role in tumorigenesis as bona fide oncogenes and tumor suppressors and as exploitable therapeutic targets.

### 4.2. Oncogenic Signaling and Protein Acetylation: Mechanistic Target of Rapamycin Complex 2 (mTORC2) as a Strong Acetylation Driver in Cancer

Accumulated evidence indicates that acetylation is an essential protein modification contributing to aggressive cancer cell phenotypes, making it important to unravel how an acetylation network is remodeled in the cancer cells in an oncogene-dependent manner. One of the major driver genes in cancer is a receptor-type tyrosine kinase (RTK), epidermal growth factor receptor (EGFR), and persistent growth factor receptor signaling including that from EGFR activates mechanistic target of rapamycin (mTOR) complex signaling, potentially affecting protein PTMs including acetylation [116]. Our recent work has demonstrated that one of the mTOR complexes, mTORC2, is a strong acetylation driver in cancer cells, especially in the context of EGFR-mutant genotypes [117].

**Figure 3:**
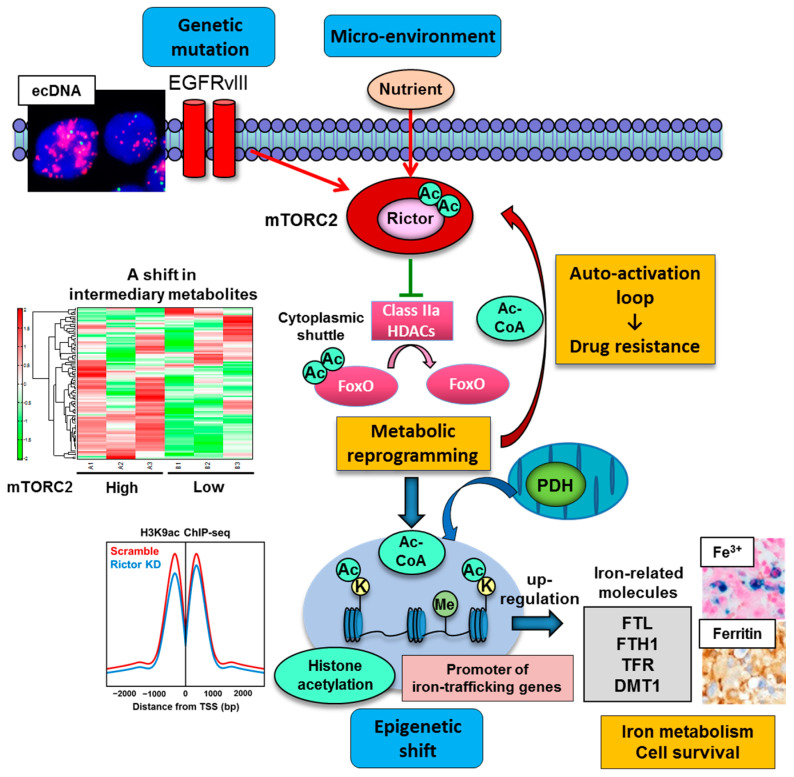
mTORC2 as a strong acetylation driver in cancer. Genetic mutation including extrachromosomal DNA (ecDNA)-dependent EGFRvIII (epidermal growth factor receptor variant III) overexpression and nutrient in the microenvironment promote mTORC2 activity which facilitates protein acetylation including cytoplasmic protein (FoxO and Rictor) and nuclear histone protein. mTORC2-dependent protein acetylation eventually contributes to c-Myc-dependent metabolic reprogramming of glycolysis, drug resistance to molecular targeting therapies, and epigenetic shift in cancer cells. Of interest, the expression of iron-related genes (FLT, FTH1, TFR, DMT1) is epigenetically promoted by mTORC2-dependent histone acetylation at their promoters, driving iron metabolism and cell survival in cancer. The findings suggest that protein acetylation driven by mTORC2 is a key player to integrate genetics, epigenetics and environment in cancer. Ac, acetyl group; Ac-CoA, acetyl-CoA; bp, base pair; ChIP-seq, chromatin immunoprecipitation sequencing; DMT1, divalent metal transporter 1; FLT, ferritin light chain; FTH1, ferritin heavy chain; FoxO, forkhead box O; HDAC, histone deacetylase; K, lysine residue; KD, knockdown; Me, methyl group; mTORC2, mechanistic target of rapamycin complex 2; PDH, pyruvate dehydrogenase; TFR, transferrin receptor; TSS, transcription start site.

We recently set out to determine the role of mTORC2 in metabolic reprogramming of the malignant brain tumor GBM, and an unexpected Akt-independent role for mTORC2 in inducing metabolic reprogramming in GBM was found [46]. mTORC2 renders GBM cells strongly addicted to glucose, and this is mediated by regulating the intracellular level of c-Myc, a crucial regulator of the Warburg effect or aerobic glycolysis. Of interest, modulation of an acetylation network of cytosolic protein by mTORC2 lies behind the cancer cell aggressiveness via metabolic reprogramming with c-Myc upregulation. mTORC2 executes an Akt-independent phosphorylation of class IIa HDACs (HDAC4, 5 and 7), which leads to the inactivating acetylation of FoxO, a negative regulator of c-Myc (Figure 3). The mechanism of FoxO inactivation relies on its acetylation to be tethered in the cytoplasm, hindering its transcriptional regulatory activity. As a result, the microRNA-dependent blockade of c-Myc is relieved, potently promoting glycolytic tumor growth. Importantly, the axis of mTORC2/acetylated FoxO/c-Myc expression confers an adverse prognostic impact to GBM patients, and it can be abrogated by dual PI3K/mTOR kinase inhibition, resulting in tumor cell death of xenograft tumor models using patient-derived GBM neurospheres. These results provide new insight into the role of mTORC2 in shaping cancer cell phenotypes through acetylation-dependent regulation of cytoplasmic and nuclear proteins (i.e., transcription factor FoxO). Additionally, an oncogenic transcription factor of c-Myc itself is known to be regulated by acetylation, providing further dimension to the acetylated web of cytoplasmic/nuclear protein interaction in cancer cells [95].

Another intriguing example of cytoplasmic protein acetylation in cancer cell phenotypes is that protein acetylation, including the acetylation of Rictor (a core component of mTORC2), can be controlled through the balance between HAT and HDAC activities [118]. We recently demonstrated that mTORC2 suppresses the activity of class IIa HDACs in EGFR-mutant GBMs through a signal cascade that results in their inactivating phosphorylation [46]. Thus, if class IIa HDACs negatively regulate mTORC2 via deacetylation of Rictor, mTORC2 can establish a feedforward auto-activation loop through inactivation of class IIa HDACs to keep Rictor in an acetylated state, maintaining downstream signaling. We demonstrated that PKCα (protein kinase C alpha) phosphorylates and inactivates class IIa HDACs downstream of mTORC2 signaling, and Rictor is in turn physically associated with class IIa HDACs and deacetylated by them [81]. This signaling cascade forms an auto-activation loop of mTORC2 and promotes the activity of mTORC2 (Figure 3). Importantly, the circuitry of mTORC2 signaling, inactivating phosphorylation of class IIa HDACs, and Rictor acetylation contributes to the resistance of cancer cells to molecular-targeting therapies [81]. Together, these results indicate that mTORC2 forms an autoactivation loop through acetyl-CoA and HDAC-mediated Rictor acetylation, which underlies the mechanism of mTORC2′s response to nutrient availability and metabolic reprogramming in EGFR-mutant GBMs (Figure 3) [11].

mTORC2 was also reported to regulate cancer epigenetics via histone acetylation, a dynamic chromatin mark with various important roles in gene regulation. Histone acetylation including H3K9ac (H3 lysine 9 acetylation) and H3K14ac (H3 lysine 14 acetylation) are controlled by mTORC2-sensitive Akt-dependent regulation of acetyl-CoA-producing enzyme ACLY [119]. In yeast, TORC2 contributes to the regulation of several histone modifications [H3K9me2 (H3 lysine 9 di-methylation), H3K4me3 (H3 lysine 4 tri-methylation) and H4K16ac (H4 lysine 16 acetylation)] for its epigenetic stability [120]. Correspondingly, our recent study demonstrates that mTORC2 promotes histone acetylation (H3K9ac, H3K18ac, H3K27ac) in the actively transcribed promoters of GBM cells through metabolic reprogramming/Warburg effect (hence the production of nuclear acetyl-CoA) and dysregulation of histone modifying enzymes including PDH and HDACs [77]. Other types of HATs (GCN5/PCAF and CBP/p300) and HDACs (HDAC3, class IIa HDACs) could contribute to the acetylation of H3K9 [117], and future studies are needed to examine whether these acetylating/deacetylating enzymes could also be regulated by mTORC2. Intriguingly, mTORC2-dependent increase in H3K9ac peaks was uniquely induced at the promoter regions of genes related to mineral metabolism including iron (Figure 3). Iron metabolism-related enzymes including ferritin light chain (FTL), ferritin heavy chain (FTH1), transferrin receptor (TFR) and divalent metal transporter 1 (DMT1) were epigenetically upregulated through histone acetylation at the promoter regions (Figure 3). Eventually, GBM cells with activated mTORC2 signaling are addicted to iron metabolism for survival, which could be therapeutically exploitable [77]. In addition to mineral metabolism, mTORC2-dependent regulation of histone H3 lysine 56 acetylation (H3K56ac) epigenetically controls the expression of glycolytic genes via regulation of sirtuin 6 (SIRT6) [121]. Together, the findings suggest that mTORC2 plays a role in the integration of cancer metabolism and PTM of the protein in an intricate, multi-directional manner, and mutual dependency of metabolism and epigenetics could be the driving force for the progression of various types of cancer, including GBM.

## 5. Novel Therapeutic Strategies to Target Protein Acetylation Systems in Cancer

**Figure 4:**
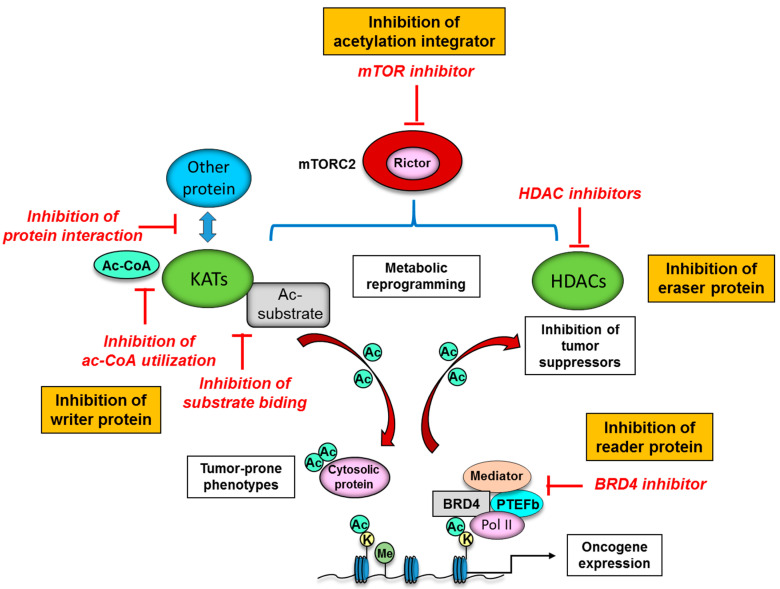
Potential therapeutic strategies to target protein acetylation systems in cancer. The interaction of KATs (writer protein) with other protein could provide specific vulnerabilities for development of small-molecule inhibitors to interfere with acetyl-CoA utilization, substrate binding and interaction with other protein such as β-catenin and HIF. Inhibition of eraser protein or HDAC can eventually reactivate the expression of tumor suppressors, resulting in cell cycle arrest, apoptosis, differentiation, and inhibition of angiogenesis and metastasis in cancer cells. BET (reader protein) inhibitors may target tumor cells without affecting normal tissues with the inhibitors’ preferential binding to super-enhancers, which are non-coding regions of DNA to bind multiple transcription factors and are critical to the expression of oncogenes. mTORC2 is an integrator of protein acetylation systems, and targeted therapies against mTORC2 could be the next-generation therapeutic strategies to interfere with cancer-specific, acetylation-dependent metabolism and epigenetics. Ac, acetyl group; Ac-CoA, acetyl-CoA; BRD4, bromodomain-containing protein 4; K, lysine residue; KAT, lysine deacetylase; HDAC, histone deacetylase; Me, methyl group; mTORC2, mechanistic target of rapamycin complex 2; PTEFb, positive transcription elongation factor b; Pol II, RNA polymerase II.

A series of novel epigenetic drug targets have been identified through the elucidation of protein acetylation mechanisms. Many KATs are not fully active unless associated with their partner proteins in KAT complexes, and integration into the complexes can affect not only the level of enzymatic activity but also substrate specificity [15]. Further, KATs themselves are subject to PTMs including acetylation that affect the activity, stability and subcellular localization [122]. Multi-layered regulation of KATs enable the control of KAT activities in an appropriate, spatio-temporal manner in the cell, but could provide specific vulnerabilities for development of small-molecule inhibitors that interfere with acetyl-CoA utilization and substrate binding (Figure 4). Such inhibition would affect acetylation of histones as well as associated transcription of oncogenes, and also have an impact on non-histone substrates, such as p53, FoxO, and c-Myc, thereby affecting the stability and activity of these transcription factors. Alternatively, small-molecule inhibitors might be designed to forestall interactions between KATs and other proteins, such as β-catenin and HIF (hypoxia-inducible factor), which would affect transcription of downstream oncogenic genes (Figure 4) [14]. However, the identification of KAT inhibitors (KATi) is not as well developed as that for HDAC inhibitors (HDACi) (Table 3).

The reversible nature of lysine acetylation by HDACs makes them promising candidates for cancer treatment targets. Indeed, the development and availability of HDACi have not only accelerated our understanding of HDAC functions and action mechanisms, but provided a promising new class of compounds for cancer treatment (Figure 4) [123,124]. HDACi has already entered into clinical trials and practical usage, and the drugs have demonstrated some effects, especially in combination with other epigenetic inhibitors [125] or chemotherapy [126]. A series of synthetic compounds and natural molecules to target class I, II, and IV HDAC enzymes have been developed and classified into four groups, including hydroxamates, benzamides, short-chain fatty acids, and cyclic peptides based on their chemical structures (Table 3) [127]. As for the actionable mechanism, the rationale for targeting HDACs in cancer is based upon the findings that altered HDAC expression and function is frequently observed in a variety of cancer types. HDACs reversibly and dynamically modify the acetylation of histone and non-histone protein, and HDACi can restore the acetylation homeostasis in cancer cells, which can eventually reactivate the expression of tumor suppressors, resulting in cell cycle arrest, apoptosis, differentiation, and inhibition of angiogenesis and metastasis (Figure 4) [128]. Of interest, cancer cells are more sensitive to HDACi-induced apoptosis than normal cells, demonstrating additional therapeutic potential of HDACi [129]. Still, the precise mechanisms by which HDACi are effective in cancer treatment await further investigation in order to select the patient who will most benefit from the treatment, reduce the side effects and induce much more potent cytotoxic effects on cancer cells.

Considering their role as readers of lysine acetylation, BET proteins are a promising target for emerging cancer therapeutics. For instance, the BET inhibitor JQ1 could displace BRD4 from chromatin and induce cell differentiation, G1 cell cycle arrest, and apoptosis in vitro as well as patient-derived xenograft models (Figure 4) [130]. In spite of peculiar pharmacologic features such as short half-life, further development of new BET inhibitors is ongoing, and the BET inhibitors were reported to downregulate the spindle checkpoint kinase [131], and cause downregulation of critical cell cycle genes as well as upregulation of cyclin dependent kinase inhibitors [132]. Even more promising, in preclinical models, BET inhibitors may target tumor cells without affecting normal tissues due to the inhibitors’ preferential binding to super-enhancers, which are non-coding regions of DNA that bind multiple transcription factors and are critical to the expression of genes that determine cellular identity [133]. BET inhibitors thus could represent potential candidates for achieving precision treatment of each cancer patient.

As for the specific role of mTORC2 in metabolic and epigenetic reprogramming, targeted therapies against mTORC2 could exemplify next-generation therapeutic strategies to interfere with cancer-specific, acetylation-dependent metabolism and epigenetics (Figure 4). However, an mTORC2-specific inhibitor is not clinically available, and it is exceptionally difficult to develop a potent and selective small-molecule inhibitor to target mTORC2 due to the intricate, multifaceted protein–protein interactions of the mTORC2 complex [134]. Novel approaches to selectively inhibit mTORC2 are emerging, which potentially provide more specific anti-cancer effects, including the targeting of complex-specific protein-protein interactions [135], and the disruption of mTORC2 substrate recruitment [136]. The demonstration that mTOR-targeting therapies could be effective cancer therapeutics through the modulation of cancer metabolism and epigenetics [137], and the future development of specific and accurate ways to inhibit mTORC2 activity represent promising strategies to target cancer metabolism and protein PTM networks.

**Table 3:** Selected KAT inhibitors and HDAC inhibitors.

KAT Inhibitors		
Mechanisms	Status	Inhibitors
Compete with substrates	Preclinical	CPTH2, CPTH6, BF1 [138,139]
Inhibit Ac-CoA utilization	Preclinical	Garcinol, C646, TH1834, Lys-CoA [140,141,142]
Block interaction with other protein	Preclinical	Chetomin (HIF), KCN1 (HIF), ICG-001 (β-catenin), Windorphen (β-catenin) [143,144,145,146]
**HDAC Inhibitors**		
**Class**	**Status**	**Inhibitors (targeted HDAC)**
Hydroxamates	FDA-approved Preclinical	* Vorinostat (SAHA) (pan-class), Belinostat (pan-class), Panobinostat (pan-class) [147,148,149] Trichostatin A (pan-class) [150]
Benzamides	Clinical trials	Entinostat (class I), Mocetinostat (class I, IV), Tacedinaline (class I) [151,152,153]
Short-chain fatty acids	Clinical trials	Valproic acid (class I, IIa), Butyric acid (class I, II), Phenylbutyrate (class I, II) [154,155,156]
Cyclic peptides	FDA-approved	Romidepsin (class I) [157]

* Combination with other epigenetic or chemotherapeutic agents could be experimentally effective in cancer models [125,126]. Ac-CoA, acetyl-CoA; HIF, hypoxia-inducible factor; KAT, lysine acetyltransferase. FDA, Food and Drug Administration; HDAC, histone deacetylase; SAHA, suberoylanilide hydroxamic acid.

## 6. Conclusions and Future Perspectives

Metabolic reprogramming is an emerging hallmark of cancer [158], and tumor development, progression and therapy response are profoundly influenced by the intracellular metabolism and the exogenous microenvironment of tumor cells, where metabolic shifts are driven by the aberration of oncogenes and tumor suppressors. Importantly, metabolic reprogramming potentially shifts the landscape of protein PTM and protein lysine acetylation lies at the interface of genetics, epigenetics and the microenvironment. In addition to the genetic aberrations themselves, the components involved in protein acetylation (i.e., writer, eraser and reader of lysine acetylation) contribute to tumorigenesis in a multifaceted fashion, implicating their importance as both regulators and effectors of aggressive cancer cell phenotypes. Recent reports suggest that the axis of metabolic reprogramming and protein modification is not unidirectional, but comprises inherently co-dependent relationships that enable tumor cells to appropriately respond to their microenvironment and ensure cell survival [4,91]. These networks enable cells to rapidly adapt to a shift in environmental nutrient condition through acetylation-dependent interaction between the promoter and enhancer regions of the survival genes. Such phenomena are well recognized in the early developmental stage of organisms, and the regulatory mechanisms could also be harnessed by cancer cells. At the same time, a slight tip in the balance of this regulation is sufficient to result in a tumor cell catastrophe. Considering that tissue context-based cues can shape metabolic dependencies [159], the “metabolic and epigenetic vulnerability” of certain cancer cells is reminiscent of the notion of “oncogene addiction” [160], the knowledge of which will lead to rational combination of cytotoxic and molecular targeted therapies [161], in order to effectively target the metabolic and epigenetic networks upon which cancer cells heavily depend. Future studies are needed to determine precisely how the primary genetic mutations specific for each tumor entity facilitate cancer metabolic reprogramming and protein modification and how, at the same time, extracellular nutrients modulate oncogenic signaling in order to translate these insights into more effective treatments for cancer patients.

## Figures and Tables

**Table 1:** Lysine acetyltransferases (KATs) family and aberration in cancer.

Family	Nomenclature	Mutation/Aberration in Cancer
GNAT family	KAT1	
	GCN5 (KAT2A)	Cancerous transformation [18]
	PCAF (KAT2B)	Missense alteration in cancer [19]
	ELP3 (KAT9)	Wnt-driven intestinal tumor initiation [20]
	ATAT-1	
	AT-1	Neurodegenerative features, inflammation and cancer [21]
	AT-2	
P300/CBP family	CBP (KAT3A)	Truncating mutations in ovarian cancer [22] Mutations and deletions in human lung cancer [23] Hematological malignancies [24]
	P300 (KAT3B)	Tumor suppressor and driver [25] Poor outcome in HNSCC [26]
MYST family	Tip60 (KAT5)	Melanoma and colon cancer [27,28]
	MOZ (KAT6A)	Association with gain-of-function p53 mutant [29] MOZ-CBP in leukemia [30]
	MORF (KAT6B)	AML [31] Leiomyoma [32]
	HBO1 (KAT7)	CSC phenotype [33]
	MOF (KAT8)	Tumor promoter in GBM [34]

AML, acute myeloid leukemia; AT-1/2, acetyl-CoA transporter 1/2; ATAT-1, alpha-tubulin *N*-acetyltransferase 1; CBP, CREB (cAMP-responsive element-binding protein)-binding protein; CSC, cancer stem cell; EPL3, elongator acetyltransferase complex subunit 3; GBM, glioblastoma; GNAT, GCN5-related N-acetyltransferases; HBO1, histone acetyltransferase binding to ORC1; HNSCC, head and neck squamous cell carcinoma; KAT, lysine acetyltransferase; MEC-17, mechanosensory abnormality 17; MOF, males absent on the first; MORF, monocytic leukemic zinc finger-related factor; MOZ, monocytic leukemia zinc finger protein; MYST, MOZ/Ybf2 (Sas3)/Sas2/Tip60; PCAF, p300/CBP-associated factor.

**Table 2:** Lysine deacetylases (KDACs/HDACs) family and aberration in caner.

Family	Nomenclature	Mutation/Aberration in Cancer
Class I	HDAC1	Mutation and CNA in DLBCL [41]
	HDAC2	MSI colon cancer [48]
	HDAC3	Liver cancer [49] gastric caner [50]
	HDAC8	Association with inv(16) fusion protein [51]
Class IIa	HDAC4	Mutation in breast cancer [52]
	HDAC5	CNA in HCC [53]
	HDAC7	Lung tumorigenesis [54]
	HDAC9	Medulloblastoma stratification [55]
Class IIb	HDAC6	Association with ARID1A-mutated ovarian cancers [56]
	HDAC10	Lung CSC phenotypes [57]
Class III (sirtuins)	SIRT1	Tumor promoter or tumor suppressor [58] Stabilization of extrachromosomal amplicons [59]
	SIRT2	DNA-damage response proteins by impairing SIRT2 catalytic activity or protein levels [60]
	SIRT3	Linked to ataxia-telangiectasia mutated (ATM) gene deficiency in DLBCL [61] HIF1α destabilization [62]
	SIRT4	Mitochondrial tumor suppressor [63]
	SIRT5	Overexpression in colorectal cancer [64]
	SIRT6	Tumor suppressor including gliomas [65]
	SIRT7	Metastatic phenotypes [66]
Class IV	HDAC11	Oncogene-induced hematopoiesis in myeloproliferative neoplasms [67]

ARID1A, AT-rich interaction domain 1A; CNA, copy number aberration; CSC, cancer stem cell; DLBCL, diffuse large B-cell lymphoma; HCC, hepatocellular carcinoma; HDAC, histone deacetylase; HIF1α, hypoxia-inducible factor 1α; MSI, microsatellite instability; SIRT, sirtuin.
